# Both Maternal and Pup Genotype Influence Ultrasonic Vocalizations and Early Developmental Milestones in *Tsc2* ^+/−^ Mice

**DOI:** 10.1155/2014/784137

**Published:** 2014-08-04

**Authors:** Emily A. Greene-Colozzi, Abbey R. Sadowski, Elyza Chadwick, Peter T. Tsai, Mustafa Sahin

**Affiliations:** The F.M. Kirby Neurobiology Center, Translational Neuroscience Center, Department of Neurology, Children's Hospital Boston, Harvard Medical School, 300 Longwood Avenue CLSB 14073, Boston, MA 02115, USA

## Abstract

Tuberous sclerosis complex (TSC) is an autosomal dominant disorder characterized by tumor growth and neuropsychological symptoms such as autistic behavior, developmental delay, and epilepsy. While research has shed light on the biochemical and genetic etiology of TSC, the pathogenesis of the neurologic and behavioral manifestations remains poorly understood. TSC patients have a greatly increased risk of developmental delay and autism spectrum disorder, rendering the relationship between the two sets of symptoms an extremely pertinent issue for clinicians. We have expanded on previous observations of aberrant vocalizations in *Tsc2* ^+/−^ mice by testing vocalization output and developmental milestones systematically during the early postnatal period. In this study, we have demonstrated that *Tsc2* haploinsufficiency in either dams or their pups results in a pattern of developmental delay in sensorimotor milestones and ultrasonic vocalizations.

## 1. Introduction

Tuberous sclerosis complex (TSC) is an autosomal dominant disease presenting with hamartomatous tumor development and neurological symptoms, including autism spectrum disorder, epilepsy, and developmental delays [1]. TSC results from mutation in either the* TSC1* or* TSC2* genes, which encode for hamartin and tuberin, respectively. These two proteins inhibit the pathway of the mammalian target of rapamycin (mTOR) through negative regulation of the GTPase Rheb [2]. The mTOR pathway plays essential roles in protein synthesis and translation that are necessary for cell proliferation [3, 4].

Approximately 25–50% of TSC patients are diagnosed with autism spectrum disorder (ASD), a developmental disorder presenting with stereotyped behavioral patterns, social impairments, and communication deficits [5]. This represents a significantly increased risk for ASD among TSC patients as compared to individuals without TSC. The neuropathology and etiology of autism remain undefined although the relationship between TSC and associated autistic symptoms continues to be explored [6]. Several studies have suggested that the neurological disruption evoked by infantile spasms as well as the presence of cortical tubers or cerebellar abnormalities increases the risk for development of ASD [7–10]. Emerging evidence from neuroimaging studies also indicates that hypomyelination, a common phenotype in the TSC brain, may be correlated with neurocognitive disabilities in patients (reviewed in [3]).

TSC patients also show an increased incidence of intellectual disability, with up to 80% of patients experiencing developmental delay [11]. In addition, epileptic symptoms associated with TSC promote risk for moderate to severe psychomotor developmental delay among patients [12]. Importantly, there is a proposed association between the type of* TSC* mutation (e.g.,* TSC1* or* TSC2*) and the risk of developmental delay, with a greater proportion of patients with* TSC2* mutations suffering from moderate to severe delay as compared to patients with* TSC1* mutations [13–15].

While most animal studies have attempted to elucidate the relationship between* TSC* and ASD, no model has completely reproduced the social impairment, communicative deficits, and stereotyped behaviors seen in autism. One particular model, the* Tsc2* ^+/−^ model, involves a nonlethal deletion of a single allele of the* Tsc2* gene [16]. These mice display a mild disease phenotype as well as neurocognitive impairments in spatial learning, both of which are reversible with rapamycin treatment [17]. Previous work on the* Tsc2* ^+/−^ model has focused on identification of an adult behavioral phenotype, but a recent study reported increased ultrasonic vocalizations (USVs) in both heterozygous and wildtype (WT) mouse pups born to* Tsc2* heterozygous dams, indicating that maternal genotype is critical for pup vocalization output [18]. However, somewhat surprisingly, this study also noted that compared to WT mothers,* Tsc2* ^+/−^ mothers displayed more vigilant maternal behavior, which might be expected to reduce the call rate of pups. Additionally, a study on USVs in the BTBR T+tf/J mouse model of autism demonstrated increased USVs in BTBR T+tf/J mice as compared to C57BL/6J WT mice, as well as abnormal development of sensorimotor milestones [19]. We therefore asked whether the previously reported association between maternal haploinsufficiency and atypical pup vocalizations is related to a more global delay in sensorimotor development. Based on previously described tests, we systematically investigated* Tsc2* ^+/−^ and WT pups from heterozygous and WT mothers for both sensory and motor development, concentrating on early development and ultrasonic vocalizations. We found increased ultrasonic vocalizations among WT pups compared to heterozygous counterparts from both WT and heterozygous mothers, indicating that pup and maternal genotype affect changes to pup vocalizations. This is also the first study to report delays in early development in the* Tsc2* ^+/−^ model that are dependent on both mother and pup genotype.

## 2. Methods

### 2.1. Animals

*Tsc2* animals from a C57BL/6J background, backcrossed for five generations (see [16]), were mated in crosses containing one heterozygous animal and one WT animal. In approximately half of the crosses, the female was heterozygous. For all tests, more than nine pups were used. Pups in each group were derived from a variety of mothers within each respective genotype group. Mice were identified using toe clippling, and date of birth was considered postnatal day (pnd) 0 for all pups. All experimental procedures were reviewed and approved by the Animal Research at Children's Hospital Boston Committee.

### 2.2. Ultrasonic Vocalizations

Beginning at pnd 4, WT and heterozygous pups from* Tsc2* WT and heterozygous dams were separated from their mother for 5 minutes and placed in a sound proof Noldus controlled acoustics chamber equipped with three microphones tuned to 50, 75, and 90 Hz. USVs were recorded using Ultravox software and were analyzed for number of calls. Testing was performed at the same time each day until pnd 10.

### 2.3. Developmental Milestones

Testing commenced on pnd 4 and continued every other day until pnd 14 for all animals. The regimen of developmental milestones included righting reflex, negative geotaxis, level screen test, cliff aversion, forelimb grasping reflex, bar holding test, and auditory startle, described in detail below [19]. Pups were weighed prior to each testing period and were reunited with their mother immediately after testing. All experiments were conducted on a heating pad (approximately 170 degrees Fahrenheit) and total daily testing lasted approximately 5 minutes per animal. Testing environment, time period, and investigator were consistent across all trials. Pup genotyping was completed after all testing had been finished in order to conduct each experiment in blinded fashion.

*Righting Reflex*. Pups were placed on their backs on a heated pad and latency to turn over (place all four paws on the surface) was recorded.

*Negative Geotaxis*. Pups were placed facing down on a piece of wire mesh (1/16′′; 8′′ × 10′′) positioned at a 45-degree angle. Latency to rotate 180 degrees and reposition facing towards the top of the screen was recorded.

*Level Screen Test*. Pups were placed on a piece of wire mesh positioned horizontally on flat surface and were gently dragged down the length of the screen by the tail. Strength of grip (measured by resistance to pulling) was recorded and scored 0 (worst) to 3 (best).

*Cliff Aversion*. Pups were positioned on wire mesh held horizontally 6 inches above the heating pad so that both front paws were hanging over the edge of the screen. Pups were scored on how rapidly they retreated from the edge of the screen (0–3).

*Bar Holding Test*. Pups were allowed to rest on a heating pad while a thin segment of wire was placed beneath the front paws. Strength of the grasping reflex was recorded and scored 0–3.

*Bar Hanging Test*. A thin segment of wire was held 6 inches above a heating pad and pups were positioned so both front paws could grasp the wire while hanging vertically. Hanging ability was scored 0–3 and was based on duration of the hang.

*Auditory Startle*. Pups were allowed to rest on the heating pad while the experimenter snapped directly behind pup's head. Startle response was recorded and scored 0–3.

### 2.4. Statistical Analysis

For all data, statistical analysis was performed using Student's* t*-test (two-way, unpaired) and Analysis of Variance (ANOVA; rep. measures). Bonferroni post-hoc tests were performed on vocalization data to identify specific points of difference. *P* < 0.05 was considered significant.

## 3. Results

### 3.1. Maternal* Tsc2* Haploinsufficiency Affects Pup Vocalizations

**Figure 1:**
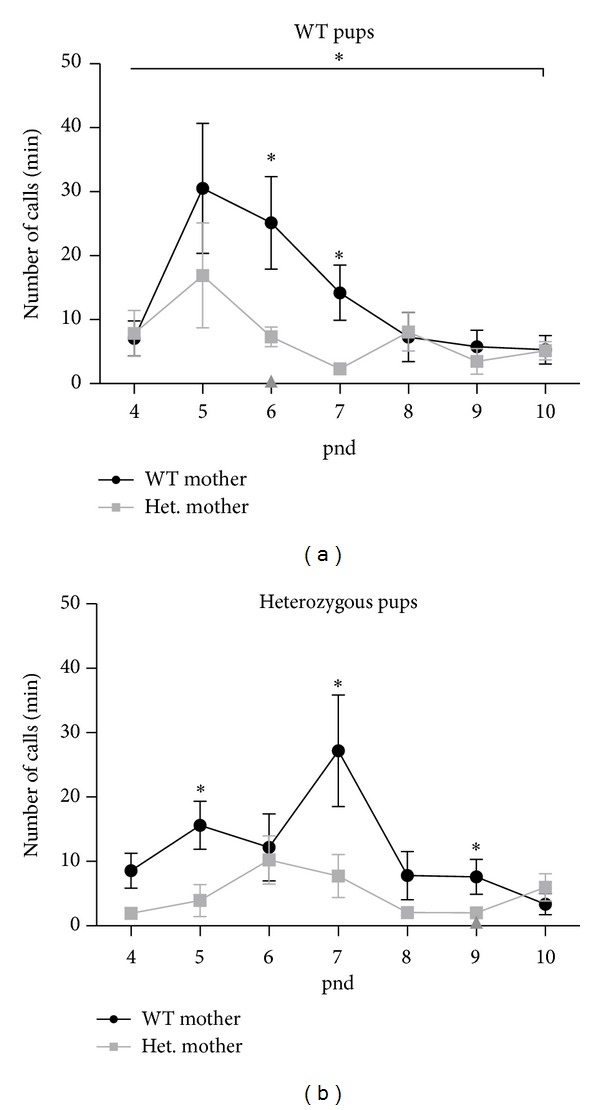
Pups born to WT or* Tsc2* heterozygous mothers emit differing numbers of calls dependent on maternal genotype. (a) WT pups from heterozygous mothers have fewer calls per minute than WT pups from WT mothers overall and at time points pnds 6 and 7. (b)* Tsc2* heterozygous pups from heterozygous mothers emit fewer calls than heterozygous pups from WT mothers at pnds 5, 7, and 9. *P < 0.05.

To evaluate early communication between mother and pups, we analyzed pup vocalizations recorded at 50–90 Hz, concentrating on rate of calls per minute. The number of calls per minute emitted by WT pups born to either* Tsc2* heterozygous or WT mothers varied by maternal genotype [*F*(1) = 12.29; *P* < 0.05] (Figure 1(a)). Bonferroni post-hoc tests identified pnd 6 as a strong point of variation (*t* = 4,1; *P* < 0.001), which was also supported with two-way* t*-tests of correlation at each time point. At pnds 6 and 7, WT pups born to* Tsc2* heterozygous dams released fewer calls than age-matched animals born to WT dams (*t*-test; *P* < 0.05; Figure 1(a)). The difference did not persist into a later age, however, and by pnd 8 WT pups from heterozygous mothers had similar call numbers to WT pups from WT mothers. All WT pups emitted a peak number of vocalizations at pnd 5, although there was a trend of pups from heterozygous mothers emitting fewer calls than counterparts from WT mothers.

Heterozygous pups from WT mothers displayed distinct USV patterns from heterozygous pups born to* Tsc2* heterozygous mothers. While the overall differences did not achieve statistical significance (*F*(1,84) = 3.26; *P* = 0.09), number of calls per minute on certain postnatal days differed according to maternal genotype. Heterozygous pups from WT mothers vocalized more than age-matched heterozygous pups from* Tsc2* heterozygous mothers at pnds 5, 7, and 9 (*t*-test; *P* < 0.05; Figure 1(b)). Heterozygous pups born to a WT mother reached their peak number of calls on pnd 7 while heterozygous pups born to heterozygous mothers had a smaller but earlier peak number of calls at pnd 6. Their mean number of calls was lower than that of matched heterozygous pups from WT mothers.

### 3.2. Maternal Haploinsufficiency Affects the Acquisition and Performance of Developmental Milestones

**Figure 2:**
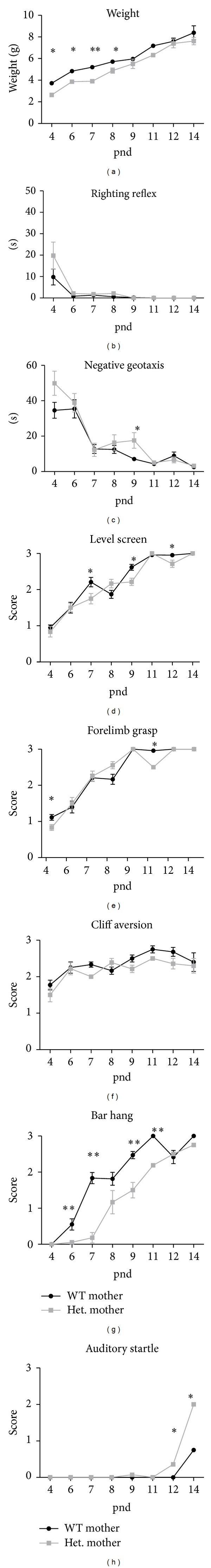
WT pups born to WT or* Tsc2* heterozygous mothers display developmental delay dependent on maternal genotype. (a) WT pups from heterozygous mothers weigh less than WT pups from WT mothers at pnds 4–8. (b) WT pups from WT and heterozygous mothers show no differences in righting reflex. (c). WT pups from heterozygous mothers delayed on negative geotaxis at pnd 9. (d) WT pups from heterozygous mothers impaired on level screen test at pnds 7, 11, and 12. (e) WT pups from heterozygous mothers show impaired forelimb grasp on pnds 4 and 11. (f) WT pups born to WT or* Tsc2* heterozygous mothers display no differences on cliff aversion. (g) WT pups from heterozygous mothers show significant impairment on bar hang at pnds 6, 7, 9, and 11. (h) WT pups from heterozygous mothers have decreased auditory startle response at pnds 12 and 14. *P < 0.05; **P < 0.005.

Since maternal genotype affected pup vocalizations, we asked whether maternal* Tsc2* genotype might also affect early sensorimotor milestone acquisition. Analysis of developmental milestones, which compared pup results based on the genotype of the dam, yielded multiple points of significant difference when WT pups from heterozygous mothers were compared to WT pups from WT mothers. WT pups from heterozygous mothers displayed delayed geotaxis at pnd 9 (*t*-test; *P* < 0.05; Figure 2(c)) and impaired reflexive grasp during the level screen test on pnds 7, 9, and 12 (*t*-test; *P* < 0.05; Figure 2(d)). On the forelimb grasp test, WT pups from heterozygous mothers displayed a decline in ability when counterparts from WT mothers were plateauing at pnd 11 (*t*-test; *P* < 0.05; Figure 2(e)) and had a similar and longer-lasting impairment in bar hang, differing significantly from controls at pnds 6, 7, 9, and 11 (*t*-test; *P* < 0.05; Figure 2(g)). In addition, they displayed an increased startle response to auditory stimulation at pnds 12 and 14 (*t*-test; *P* < 0.05; Figure 2(h)). No difference in the righting reflex or cliff aversion was detected (Figures 2(b) and 2(f)). With the exception of the auditory startle test, all observed delays were not permanent and did not persist into the final day of testing (pnd 14). WT pups from heterozygous mothers weighed less than their WT counterparts from WT mothers, starting at pnd 4, continuing to be underweight until pnd 8 (*t*-test; *P* < 0.05; Figure 2(a)). We analyzed the other milestones from pnds 4–8 to determine if the underweight animals displayed similarly timed delays in development, which might indicate an effect of weight rather than genotype, and observed that weight differences did not correlate with any significant delays.

**Figure 3:**
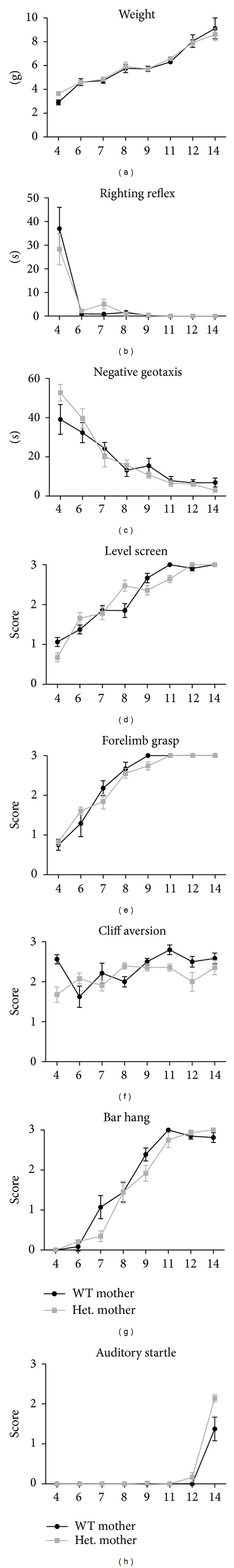
*Tsc2* heterozygous pups from WT or heterozygous mothers display no significant difference in developmental milestones.

The effect of maternal genotype on developmental milestones in heterozygous pups was also examined. However, when statistical comparisons were made between all heterozygous pups born to WT and heterozygous mothers, no significant differences were observed based on maternal genotype (Figures 3(a)–3(h)).

### 3.3. Pup* Tsc2* Haploinsufficiency Affects Vocalizations

**Figure 4:**
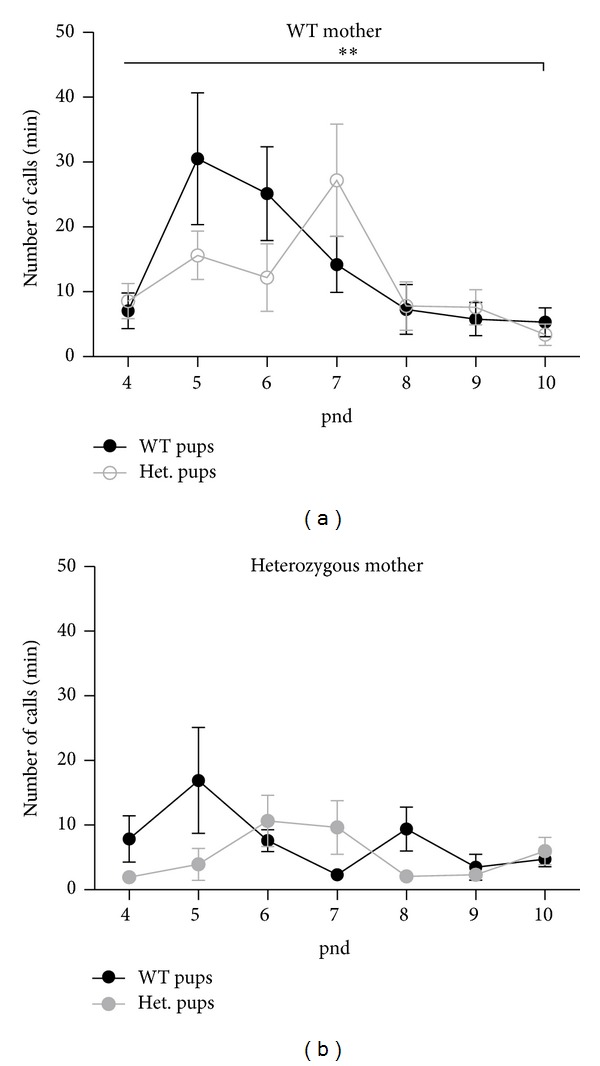
WT and* Tsc2* heterozygous pups born to WT or* Tsc2* heterozygous mothers display differences in overall number of calls dependent on pup genotype. (a) Heterozygous pups from WT mothers have delayed peak number of vocalizations. (b) WT and heterozygous pups from* Tsc2* heterozygous mothers show no significant differences in number of calls; although not statistically relevant, heterozygous pups have delayed peak number of calls. **P < 0.005.

Although our and previous results show that maternal genotype is critical in determining pup vocalizations, we also wanted to investigate the impact of pup genotype on vocalizations and development. While previous studies did not observe an effect of pup genotype on USVs, we analyzed our data for an effect of pup genotype and discovered significant differences parallel to those observed for maternal genotype. WT pups (from WT dams) displayed a peak number of <30 calls per minute on pnd 5. The number of calls subsequently declined until pnd 9, when they reached a plateau of >10 calls per minute (Figure 4(a)).* Tsc2* heterozygous pups from WT mothers emitted fewer calls than WT littermates at pnds 5 and 6 and reached their peak number of calls per minute at pnd 7 at a time when WT littermates displayed a marked decrease in calling (Figure 4(a)). The differences observed between WT and heterozygous pups from WT mothers failed to reach statistical significance at specific time points; however, a repeated measures analysis of variance showed significant difference in number of calls with an overall effect of genotype over the entire multiday testing period [*F*(13) = 4.06; *P* < 0.001].

Pups from heterozygous mothers also displayed distinct vocalization patterns based on their genotype (Figure 4(b)). As with WT mothers, heterozygous pups showed a trend towards a later and smaller peak number of vocalizations than WT littermates, reaching their maximum number of calls at pnd 7, as opposed to WT pups, which reached maximum calls at pnd 5. These differences however did not reach statistical significance, likely related to baseline low levels of calls in all pups born to heterozygous mothers.

### 3.4. Pup* Tsc2* Haploinsufficiency Affects the Acquisition and Performance of Developmental Milestones

**Figure 5:**
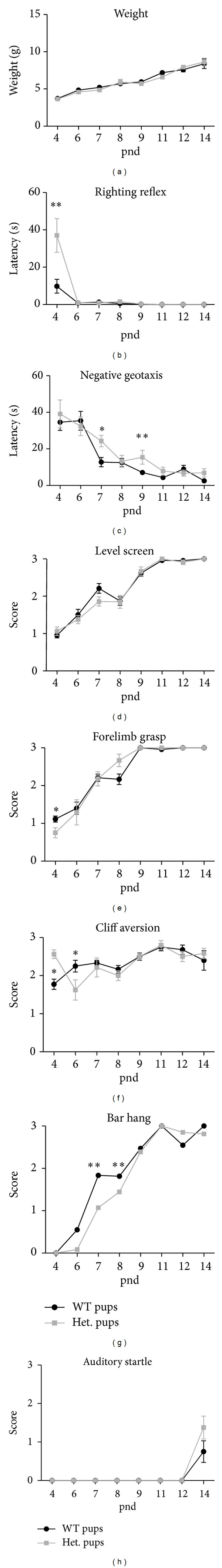
WT and* Tsc2* heterozygous pups born to WT mothers display delayed development for certain milestones. (a) WT and heterozygous pups have no differences in weight. (b)* Tsc2* heterozygous pups from WT mothers display delayed righting reflex compared to WT pups at pnd 4. (c) Heterozygous pups have delayed geotaxis at pnds 7 and 9 as compared to WT pups. (d) WT and heterozygous pups show no significant differences in forelimb grasp. (e) WT and heterozygous pups show no significant differences in level screen test. (f) WT and heterozygous pups show no significant differences in cliff aversion. (g) Heterozygous pups have impaired bar hang as compared to WT pups at pnds 7 and 8. (h) WT and heterozygous pups have no significant differences in auditory startle response. *P < 0.05; **P < 0.005.

**Figure 6:**
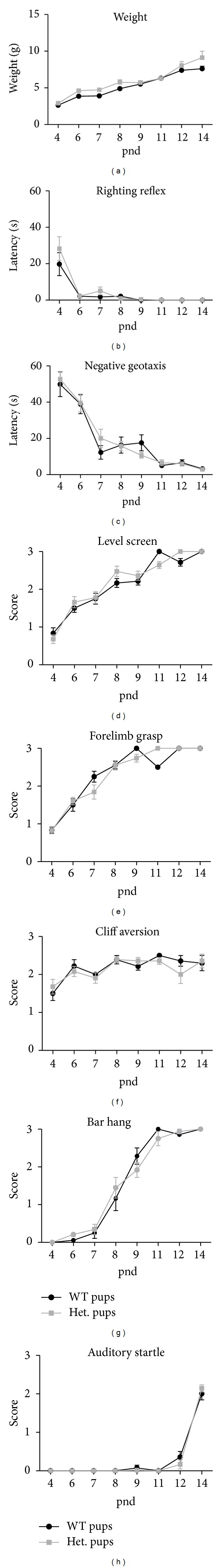
WT and* Tsc2* heterozygous pups born to* Tsc2* heterozygous mothers display no significant differences in developmental milestones.

Since pup genotype affected vocalizations in pups born to WT mothers, we then analyzed the milestone data for an effect of pup genotype and found that heterozygous and WT pups from WT mothers (Figure 5) displayed greater differences in their performance of developmental tests than did heterozygous and WT pups from a heterozygous mother (Figure 6). WT and heterozygous pups with consistent WT maternal genotype showed statistically significant differences in righting reflex, negative geotaxis, forelimb grasp, cliff aversion, and bar hang (Figures 5(a)–5(d) and 5(f)-5(g)). Most differences manifested only between pnds 4 and 9, suggesting that the developmental delay is early and temporary. At pnd 4, heterozygous pups from a WT mother showed delayed righting reflex (*t*-test; *P* < 0.05; Figure 5(b)) and impaired forelimb grasping reflex and cliff aversion (*t*-test; *P* < 0.05; Figures 5(e) and 5(f)) as compared to WT littermates. Cliff aversion was also impaired at pnd 6 (*t*-test; *P* < 0.05; Figure 5(f)), but that difference did not persist past this time point. At pnd 7, heterozygous pups demonstrated a delay in latency to complete negative geotaxis (*t*-test; *P* < 0.05; Figure 5(c)) and impairment in bar hanging (*t*-test; *P* < 0.05; Figure 5(g)) when compared to WT littermates. Heterozygous pups displayed continued bar hang impairment at pnd 8 (*t*-test; *P* < 0.05; Figure 5(g)) and showed a delay in latency to negative geotaxis during that time point (*t*-test; *P* < 0.05; Figure 5(b)). After pnd 9, heterozygous and WT pups had comparable results for all tests excluding negative geotaxis, which remained delayed in heterozygous pups until pnd 11 (*t*-test; *P* < 0.05; Figure 5(b)).

Heterozygous and WT pups born to heterozygous mothers were closely matched in performance and acquisition of all milestones, likely due to a strong effect of maternal haploinsufficiency. No significant differences were noted in developmental milestone tests based on pup genotype (Figures 6(a)–6(h)).

## 4. Discussion

USVs, which range from 50 to 90 Hz, are reliable indicators of the mother-pup relationship and of early pup development. They begin 3-4 days after birth and reach a peak between 6 and 8 days [20]. Socially, they enable the juvenile pups to communicate with their mother during and after separation by eliciting searching behavior during separation and retrieval during reunion. Although the possible role of pup vocalizations in communication is still being investigated, it has been shown that vocalizations by juvenile pups do elicit searching behavior in the mother during periods of separation [21]. Vocalizations also appear to be dependent on mouse strain, suggesting that they are sensitive to genetic differences [22]. A previous study showed increases in* Tsc2* ^+/−^ pup vocalizations based on maternal genotype and reported greater maternal attention from heterozygous mothers [18]. Our results are congruent with previous reports of increased maternal attention from heterozygous mothers: pups born to heterozygous mothers emit fewer distress calls than those born to WT mothers, suggesting that heterozygous mothers display more vigilant search and retrieval behavior. Fewer pup calls generally indicate increased maternal vigilance with resulting reductions in pup calls. Our reports of decreased numbers of vocalizations based on pup and maternal heterozygosity differ from the observations published by Young and colleagues in 2010, which presented evidence that WT pups from heterozygous dams vocalized more; that is, they were potentiated after isolation. One possible explanation for this discrepancy is the difference in experimental design: our methods closely followed those detailed in Scattoni's multiday study of the BTBR mice [19] while Young and colleagues focused on pups at pnd 10. Nonetheless, our results are consistent with their reports of increased maternal attention in heterozygote mothers.

We show a novel effect of both maternal and pup haploinsufficiency on vocalizations and also report a trend of developmental delay dependent on haploinsufficiency. TSC is associated with high instance of developmental delay and autistic phenotype; previous studies looking at early development and vocalizations in* Tsc2* ^+/−^ mice found that maternal heterozygosity at the* Tsc2* locus is linked to changes in duration and number of calls per minute of pup USVs [18]. However, our results shed new light on the relationship between* Tsc2* haploinsufficiency and risk for early developmental delay both in sensorimotor development and in communication. In all genotypic scenarios (*Tsc2* haploinsufficiency in either dam or pup), heterozygosity had a consistently suppressive effect on either production of USVs or performance of sensorimotor milestones. All pups with heterozygous mothers, regardless of individual genotype, produced reduced numbers of USVs than age-matched counterparts from WT mothers or showed a delay in peak number of calls. Additionally, all heterozygous pups from either WT or heterozygous mothers displayed a delay in peak number of calls when compared to WT pups. The greatest overall differences in calls per minute arise among WT pups from WT dams and counterparts with any heterozygosity: maternal, pup, or both. This suggests further evidence that haploinsufficiency, whether contributing to a behavioral phenotype in the mother or causing intrinsic delays in pup sensorimotor development, may exert a suppressive force on pup development strong enough to prevent significant differences among heterozygous pups and pups from heterozygous mothers.

While USVs analysis provides convincing evidence for socially evoked vocalization abnormalities being due to maternal genotype, both developmental milestones and vocalizations, when analyzed for an effect of pup genotype, provide evidence for additional developmental delay that is dependent on pup, rather than dam, genotype. Heterozygous pups born to WT mothers showed delayed righting reflex and geotaxis as well as impaired bar holding and grasping skills when compared to WT littermates, and WT pups from heterozygous dams displayed similarly consistent delays across tests. Taken together, these observations indicate that the delay observed during the sensorimotor and vocalization testing is not only a consequence of differential maternal behavior, as previously suggested [18], but also an autonomous genetically determined phenotype. However, the behavioral phenotype of the pups likely arises from a combination of genetic predisposition and the mother-pup relationship rather than occurring as a sole consequence of environment or genes only.

We find that maternal genotype plays a determining role in the number of pup vocalizations while pup genotype affects the timing of the peak number of calls per minute, delaying it in the case of pup heterozygosity. Maternal heterozygosity also has an effect on pup sensorimotor development, causing delays especially in WT offspring. Additionally, pup heterozygosity delays acquisition of certain developmental skills among pups born to WT dams. These findings indicate a strong genetic relationship between* Tsc2* heterozygosity and developmental delay, providing further evidence that the* Tsc2* mutation is associated with impaired development, as has been previously suggested clinically [23]. Loss of both alleles of* Tsc1* or* Tsc2* in mice impairs neuronal migration [24, 25], while haploinsufficiency leads to disrupted connectivity between retinal neurons and their thalamic targets [26].* Tsc1* or* Tsc2* mutant animals also have reduced CNS myelination [25, 27]. Thus far, the* Tsc2* ^*+/*−^ mouse has demonstrated deficits in hippocampal-dependent learning and social communication without the presence of the epileptic seizures that are proposed to trigger these neuropsychological symptoms [17, 18]. The deficits, as evidenced by the developmental milestone tests, show that the atypical pup USVs in this animal model are strongly linked to an intrinsic condition and environmental factors. Of note, it is widely thought that epilepsy itself may significantly contribute to psychomotor delay in humans. There are other mouse models of TSC that display spontaneous seizures and can be used to investigate the relationship between epilepsy and psychomotor delay. Future investigation into and characterization of these delayed skills will be essential in elucidating the complete behavioral phenotype of the* Tsc2* ^*+/*−^ mice and other TSC animal models. While the mechanistic association between the disrupted neuronal pathways and the manifestation of neurologic symptoms is still under investigation, the high instance of such symptoms among TSC patients necessitates further research, and this study provides a method to investigate the pathogenesis of early developmental delay and the ASD phenotype.
